# Supplementary material for: Diethyl Blechnic Exhibits Anti-Inflammatory and Antioxidative Activity via the TLR4/MyD88 Signaling Pathway in LPS-Stimulated RAW264.7 Cells
Source: Molecules. 2019 Dec 9;24(24):4502. doi: 10.3390/molecules24244502 (PMC6943418; doi:10.3390/molecules24244502)
Supplement: Supplementary file 1 [file molecules-24-04502-s001.pdf]

Diethyl Blechnic exhibits anti-inflammatory and antioxidative activity *via* the TLR4/MyD88 Signaling Pathway in LPS-stimulated RAW264.7 Cells

Jia He[1,2], Shan Han[1,2], Xin-xing Li[1,2], Qin-qin Wang[1,2], Yushun Cui[3], Yangling Chen[1,2], Hongwei Gao[1,2*], Liting Huang[1,2*], Shilin Yang[1,2]

[1] College of Pharmacy, Guangxi University of Chinese Medicine, Nanning 530000, China; [2] Guangxi Engineering Technology Research Center of Advantage Chinese Patent Drug and Ethnic Drug Development, Nanning, 530200; [3] State Key Laboratory of Innovative Drug and Efficient Energy-Saving Pharmaceutical Equipment, Jiangxi University of Traditional Chinese Medicine, Nanchang 330004, China.

Running Title: Diethyl Blechnic exerts anti-inflammatory and antioxidative effects.

Co-corresponding Authors:

Dr. Hongwei Gao; Email: gaohongwei06@126.com; Address: College of Pharmacy, Guangxi University of Chinese Medicine, Nanning 530000, China; Nanning 530000, China; Tel: +86-13878876311 Fax: +86-771-4953513.

Ms. Liting Huang; Email:huangliting2007happy@126.com; Address: College of Pharmacy, Guangxi University of Chinese Medicine, Nanning 530000, China; Tel: +86-15277171950 Fax: +86-771-4953513.

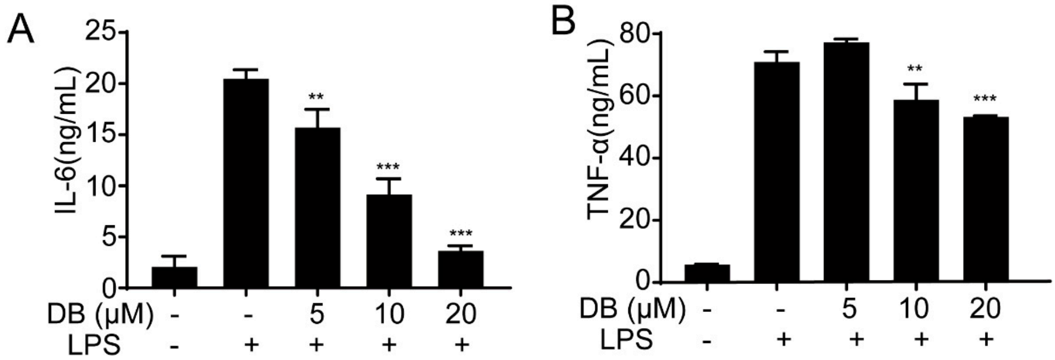

Figure S1. DB suppressed the release of pro-inflammatory cytokines in LPS-induced RAW264.7 cells. RAW 264.7 cells were pretreated with DB for 1 h and then stimulated with LPS (1 μg/mL) for 24 h. The levels of IL-6 (**A**) and TNF-α (**B**) in the culture medium were determined by ELISA kits. (n=3). **p< 0.01, and ***p< 0.001 vs LPS group.

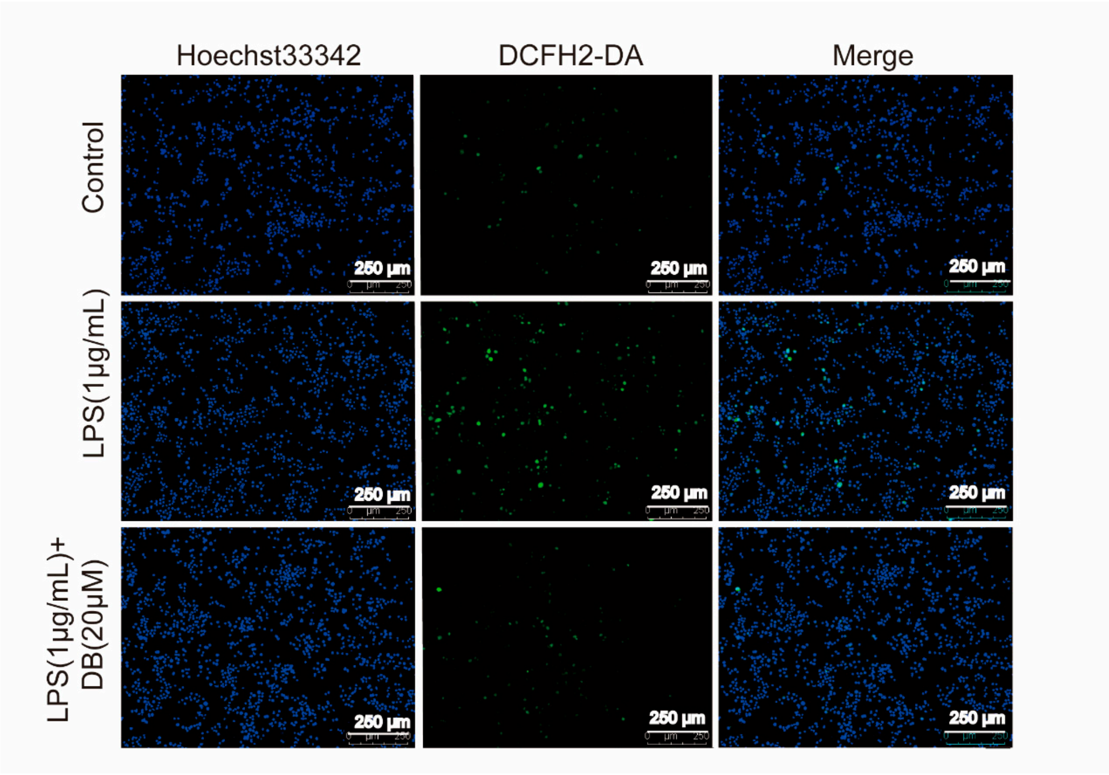

Figure S2. DB suppressed LPS-induced ROS generation in RAW264.7 cells. Cells pretreated with indicated concentrations of DB for 1 h were co-cultured with LPS (1 μg/mL) for another 6 h. Then cells labeled with $DCFH_2$-DA (10 μM) for 30 min were investigated by a fluorescence microscope, (n=3). The scale bar, 250 μm.

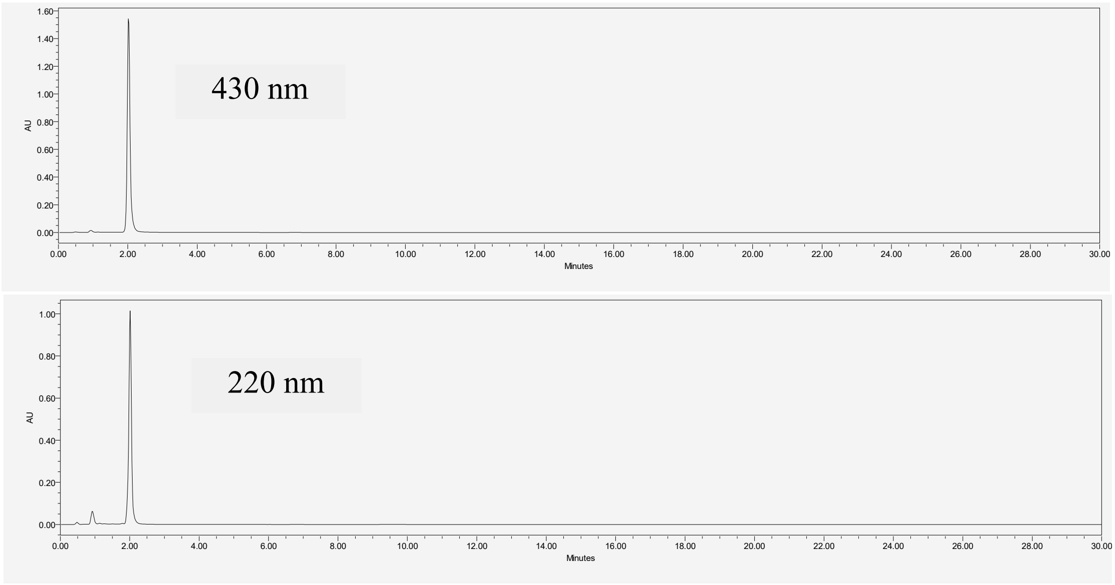

Figure S3. The chromatographic of DB. The HPLC condtions:the mobile flow phase: acetonitrile/water (50/50), the flow rate: 1 mL/min, the wave length: 430 and 220 nm.
